# Supplementary material for: Modeling glioblastoma heterogeneity as a dynamic network of cell states
Source: Mol Syst Biol. 2021 Sep 16;17(9):e10105. doi: 10.15252/msb.202010105 (PMC8444284; doi:10.15252/msb.202010105)
Supplement: Supplementary file 6 — Source Data for Figure 5 [file MSB-17-e10105-s004.zip › Figure5A_sourcedata/GSEA_3017/nosigdb_stateE.GseaPreranked.1622795031093/FELDMAN_2020_S-G2.html]

Details for gene set FELDMAN\_2020\_S-G2[GSEA]

|  || Dataset | state63017 |
| Phenotype | NoPhenotypeAvailable |
| Upregulated in class | na\_neg |
| GeneSet | FELDMAN\_2020\_S-G2 |
| Enrichment Score (ES) | -0.59805423 |
| Normalized Enrichment Score (NES) | -3.5039172 |
| Nominal p-value | 0.0 |
| FDR q-value | 0.0 |
| FWER p-Value | 0.0 |
Table: GSEA Results Summary

  

Fig 1: Enrichment plot: FELDMAN\_2020\_S-G2      
 Profile of the Running ES Score & Positions of GeneSet Members on the Rank Ordered List

  

| PROBE | GENE SYMBOL | GENE\_TITLE | RANK IN GENE LIST | RANK METRIC SCORE | RUNNING ES | CORE ENRICHMENT || 1 | CTGF |  |  | 2 | 1.276 | 0.0070 | No |
| 2 | IGFBP5 |  |  | 4 | 1.015 | 0.0134 | No |
| 3 | ID3 |  |  | 6 | 0.972 | 0.0195 | No |
| 4 | FABP7 |  |  | 11 | 0.920 | 0.0211 | No |
| 5 | LYPD1 |  |  | 13 | 0.813 | 0.0259 | No |
| 6 | TTYH1 |  |  | 19 | 0.731 | 0.0247 | No |
| 7 | IFITM3 |  |  | 26 | 0.682 | 0.0217 | No |
| 8 | CDH2 |  |  | 27 | 0.668 | 0.0268 | No |
| 9 | ENC1 |  |  | 58 | 0.501 | -0.0101 | No |
| 10 | UPP1 |  |  | 78 | 0.438 | -0.0326 | No |
| 11 | BEX1 |  |  | 88 | 0.419 | -0.0417 | No |
| 12 | NDFIP1 |  |  | 91 | 0.416 | -0.0412 | No |
| 13 | GPM6B |  |  | 105 | 0.391 | -0.0559 | No |
| 14 | CLU |  |  | 119 | 0.364 | -0.0708 | No |
| 15 | ARL6IP5 |  |  | 134 | 0.345 | -0.0873 | No |
| 16 | DCBLD2 |  |  | 165 | 0.322 | -0.1256 | No |
| 17 | CALM2 |  |  | 191 | 0.303 | -0.1573 | No |
| 18 | SAT1 |  |  | 206 | 0.291 | -0.1741 | No |
| 19 | TNFRSF12A |  |  | 262 | 0.257 | -0.2470 | No |
| 20 | DONSON |  |  | 274 | -0.251 | -0.2601 | No |
| 21 | SUPT16H |  |  | 276 | -0.251 | -0.2595 | No |
| 22 | RMI2 |  |  | 292 | -0.257 | -0.2779 | No |
| 23 | RAN |  |  | 298 | -0.257 | -0.2828 | No |
| 24 | WDR34 |  |  | 303 | -0.258 | -0.2862 | No |
| 25 | HNRNPAB |  |  | 312 | -0.261 | -0.2951 | No |
| 26 | HMGA1 |  |  | 313 | -0.261 | -0.2931 | No |
| 27 | PSIP1 |  |  | 319 | -0.263 | -0.2979 | No |
| 28 | HNRNPDL |  |  | 322 | -0.263 | -0.2986 | No |
| 29 | TUBG1 |  |  | 323 | -0.263 | -0.2966 | No |
| 30 | MAGOHB |  |  | 328 | -0.264 | -0.3001 | No |
| 31 | HSPD1 |  |  | 329 | -0.264 | -0.2980 | No |
| 32 | PAICS |  |  | 336 | -0.265 | -0.3042 | No |
| 33 | RPL22L1 |  |  | 350 | -0.269 | -0.3198 | No |
| 34 | NUDT15 |  |  | 356 | -0.270 | -0.3246 | No |
| 35 | POP7 |  |  | 370 | -0.273 | -0.3402 | No |
| 36 | TMEM237 |  |  | 383 | -0.276 | -0.3544 | No |
| 37 | HSPA8 |  |  | 392 | -0.280 | -0.3631 | No |
| 38 | SNRPG |  |  | 398 | -0.283 | -0.3678 | No |
| 39 | DERA |  |  | 402 | -0.284 | -0.3697 | No |
| 40 | LSM4 |  |  | 406 | -0.284 | -0.3716 | No |
| 41 | NASP |  |  | 410 | -0.285 | -0.3735 | No |
| 42 | SIVA1 |  |  | 412 | -0.286 | -0.3727 | No |
| 43 | CHCHD2 |  |  | 423 | -0.290 | -0.3841 | No |
| 44 | KPNB1 |  |  | 445 | -0.298 | -0.4104 | No |
| 45 | ACYP1 |  |  | 454 | -0.301 | -0.4189 | No |
| 46 | HNRNPD |  |  | 456 | -0.301 | -0.4180 | No |
| 47 | SNRPE |  |  | 458 | -0.302 | -0.4171 | No |
| 48 | DNMT1 |  |  | 466 | -0.304 | -0.4243 | No |
| 49 | SMC1A |  |  | 472 | -0.307 | -0.4287 | No |
| 50 | POLR3K |  |  | 474 | -0.307 | -0.4277 | No |
| 51 | RFC2 |  |  | 498 | -0.313 | -0.4566 | No |
| 52 | PA2G4 |  |  | 501 | -0.314 | -0.4570 | No |
| 53 | GGH |  |  | 504 | -0.314 | -0.4573 | No |
| 54 | PGP |  |  | 512 | -0.318 | -0.4644 | No |
| 55 | HAUS1 |  |  | 518 | -0.320 | -0.4687 | No |
| 56 | SESN3 |  |  | 526 | -0.322 | -0.4758 | No |
| 57 | RHEB |  |  | 528 | -0.323 | -0.4747 | No |
| 58 | RFC5 |  |  | 534 | -0.326 | -0.4790 | No |
| 59 | RBM17 |  |  | 546 | -0.331 | -0.4915 | No |
| 60 | MCM3 |  |  | 560 | -0.340 | -0.5065 | No |
| 61 | HAT1 |  |  | 561 | -0.340 | -0.5039 | No |
| 62 | TCF19 |  |  | 569 | -0.342 | -0.5109 | No |
| 63 | CKLF |  |  | 574 | -0.347 | -0.5137 | No |
| 64 | CACYBP |  |  | 589 | -0.352 | -0.5300 | No |
| 65 | EIF4EBP1 |  |  | 592 | -0.353 | -0.5300 | No |
| 66 | CMSS1 |  |  | 593 | -0.353 | -0.5273 | No |
| 67 | HSPB11 |  |  | 594 | -0.354 | -0.5246 | No |
| 68 | GGCT |  |  | 598 | -0.356 | -0.5260 | No |
| 69 | RNASEH2B |  |  | 599 | -0.356 | -0.5233 | No |
| 70 | HIRIP3 |  |  | 603 | -0.359 | -0.5246 | No |
| 71 | ZWILCH |  |  | 604 | -0.360 | -0.5219 | No |
| 72 | PARP2 |  |  | 609 | -0.362 | -0.5245 | No |
| 73 | BTG3 |  |  | 615 | -0.365 | -0.5286 | No |
| 74 | HNRNPF |  |  | 621 | -0.369 | -0.5326 | No |
| 75 | EXOSC8 |  |  | 630 | -0.376 | -0.5406 | No |
| 76 | RNASEH2A |  |  | 633 | -0.378 | -0.5404 | No |
| 77 | CDKN2D |  |  | 638 | -0.381 | -0.5429 | No |
| 78 | TMEM106C |  |  | 654 | -0.397 | -0.5603 | No |
| 79 | SHCBP1 |  |  | 655 | -0.398 | -0.5573 | No |
| 80 | GINS2 |  |  | 658 | -0.399 | -0.5569 | No |
| 81 | DCK |  |  | 659 | -0.400 | -0.5539 | No |
| 82 | HIST1H2AM |  |  | 660 | -0.400 | -0.5508 | No |
| 83 | SAE1 |  |  | 668 | -0.408 | -0.5572 | No |
| 84 | DSN1 |  |  | 671 | -0.410 | -0.5568 | No |
| 85 | NUDT1 |  |  | 674 | -0.413 | -0.5564 | No |
| 86 | SAC3D1 |  |  | 679 | -0.416 | -0.5587 | No |
| 87 | H2AFV |  |  | 684 | -0.423 | -0.5609 | No |
| 88 | CDCA4 |  |  | 687 | -0.424 | -0.5603 | No |
| 89 | SLC25A5 |  |  | 695 | -0.426 | -0.5666 | No |
| 90 | DIAPH3 |  |  | 696 | -0.427 | -0.5634 | No |
| 91 | RANBP1 |  |  | 697 | -0.427 | -0.5601 | No |
| 92 | ANP32B |  |  | 699 | -0.431 | -0.5582 | No |
| 93 | TEX30 |  |  | 709 | -0.436 | -0.5671 | No |
| 94 | MZT1 |  |  | 711 | -0.437 | -0.5651 | No |
| 95 | RBBP8 |  |  | 714 | -0.440 | -0.5645 | No |
| 96 | C19orf48 |  |  | 738 | -0.462 | -0.5922 | No |
| 97 | SMC3 |  |  | 743 | -0.470 | -0.5941 | No |
| 98 | PTMA |  |  | 745 | -0.475 | -0.5918 | No |
| 99 | TK1 |  |  | 747 | -0.480 | -0.5895 | No |
| 100 | MIS18A |  |  | 750 | -0.483 | -0.5885 | No |
| 101 | SPDL1 |  |  | 758 | -0.491 | -0.5943 | Yes |
| 102 | CDCA3 |  |  | 759 | -0.497 | -0.5905 | Yes |
| 103 | TRIP13 |  |  | 760 | -0.498 | -0.5867 | Yes |
| 104 | MXD3 |  |  | 763 | -0.502 | -0.5856 | Yes |
| 105 | GMNN |  |  | 766 | -0.510 | -0.5844 | Yes |
| 106 | RTKN2 |  |  | 767 | -0.511 | -0.5805 | Yes |
| 107 | LRR1 |  |  | 768 | -0.512 | -0.5766 | Yes |
| 108 | CMC2 |  |  | 771 | -0.518 | -0.5754 | Yes |
| 109 | BTG1 |  |  | 772 | -0.520 | -0.5714 | Yes |
| 110 | CDC45 |  |  | 773 | -0.521 | -0.5674 | Yes |
| 111 | DTYMK |  |  | 774 | -0.522 | -0.5634 | Yes |
| 112 | RFC4 |  |  | 777 | -0.527 | -0.5621 | Yes |
| 113 | STMN1 |  |  | 778 | -0.529 | -0.5581 | Yes |
| 114 | CENPA |  |  | 782 | -0.538 | -0.5580 | Yes |
| 115 | SKA3 |  |  | 783 | -0.539 | -0.5539 | Yes |
| 116 | RPA3 |  |  | 788 | -0.550 | -0.5552 | Yes |
| 117 | ATAD5 |  |  | 791 | -0.559 | -0.5536 | Yes |
| 118 | HMGN2 |  |  | 795 | -0.564 | -0.5534 | Yes |
| 119 | HMGB1 |  |  | 797 | -0.567 | -0.5504 | Yes |
| 120 | ASF1B |  |  | 798 | -0.568 | -0.5461 | Yes |
| 121 | TUBB |  |  | 802 | -0.570 | -0.5458 | Yes |
| 122 | CEP78 |  |  | 803 | -0.570 | -0.5415 | Yes |
| 123 | TUBB4B |  |  | 804 | -0.573 | -0.5371 | Yes |
| 124 | CENPN |  |  | 805 | -0.574 | -0.5327 | Yes |
| 125 | USP1 |  |  | 806 | -0.575 | -0.5283 | Yes |
| 126 | PSMC3IP |  |  | 811 | -0.588 | -0.5293 | Yes |
| 127 | DEK |  |  | 812 | -0.591 | -0.5248 | Yes |
| 128 | DEPDC1 |  |  | 815 | -0.596 | -0.5229 | Yes |
| 129 | CDCA2 |  |  | 816 | -0.597 | -0.5184 | Yes |
| 130 | MCM7 |  |  | 817 | -0.599 | -0.5138 | Yes |
| 131 | LMNB1 |  |  | 818 | -0.606 | -0.5091 | Yes |
| 132 | MND1 |  |  | 821 | -0.615 | -0.5072 | Yes |
| 133 | CKAP2L |  |  | 826 | -0.625 | -0.5078 | Yes |
| 134 | CCDC34 |  |  | 827 | -0.626 | -0.5031 | Yes |
| 135 | DNAJC9 |  |  | 828 | -0.633 | -0.4982 | Yes |
| 136 | AURKB |  |  | 829 | -0.634 | -0.4934 | Yes |
| 137 | HELLS |  |  | 830 | -0.635 | -0.4885 | Yes |
| 138 | PCNA |  |  | 832 | -0.640 | -0.4850 | Yes |
| 139 | BUB3 |  |  | 834 | -0.641 | -0.4815 | Yes |
| 140 | CENPW |  |  | 835 | -0.643 | -0.4766 | Yes |
| 141 | KIF22 |  |  | 842 | -0.678 | -0.4795 | Yes |
| 142 | BIRC5 |  |  | 843 | -0.679 | -0.4744 | Yes |
| 143 | CENPM |  |  | 844 | -0.682 | -0.4692 | Yes |
| 144 | ZWINT |  |  | 847 | -0.692 | -0.4666 | Yes |
| 145 | CENPH |  |  | 849 | -0.694 | -0.4626 | Yes |
| 146 | BRCA2 |  |  | 850 | -0.694 | -0.4573 | Yes |
| 147 | DHFR |  |  | 851 | -0.701 | -0.4520 | Yes |
| 148 | VRK1 |  |  | 852 | -0.711 | -0.4466 | Yes |
| 149 | NUF2 |  |  | 856 | -0.721 | -0.4451 | Yes |
| 150 | CENPK |  |  | 857 | -0.722 | -0.4396 | Yes |
| 151 | SYNE2 |  |  | 859 | -0.728 | -0.4354 | Yes |
| 152 | ANLN |  |  | 860 | -0.733 | -0.4298 | Yes |
| 153 | KIF4A |  |  | 861 | -0.736 | -0.4242 | Yes |
| 154 | FEN1 |  |  | 862 | -0.736 | -0.4186 | Yes |
| 155 | FBXO5 |  |  | 864 | -0.742 | -0.4143 | Yes |
| 156 | RRM1 |  |  | 866 | -0.749 | -0.4099 | Yes |
| 157 | ARHGAP11A |  |  | 867 | -0.756 | -0.4041 | Yes |
| 158 | NDC80 |  |  | 869 | -0.762 | -0.3997 | Yes |
| 159 | ORC6 |  |  | 870 | -0.766 | -0.3938 | Yes |
| 160 | CCNA2 |  |  | 872 | -0.770 | -0.3893 | Yes |
| 161 | KIF23 |  |  | 873 | -0.772 | -0.3834 | Yes |
| 162 | MELK |  |  | 874 | -0.776 | -0.3775 | Yes |
| 163 | TMPO |  |  | 875 | -0.778 | -0.3715 | Yes |
| 164 | KIF20B |  |  | 876 | -0.779 | -0.3656 | Yes |
| 165 | CEP55 |  |  | 877 | -0.780 | -0.3596 | Yes |
| 166 | CKAP2 |  |  | 878 | -0.781 | -0.3537 | Yes |
| 167 | PTTG1 |  |  | 880 | -0.791 | -0.3490 | Yes |
| 168 | H2AFX |  |  | 882 | -0.799 | -0.3442 | Yes |
| 169 | KIFC1 |  |  | 883 | -0.807 | -0.3381 | Yes |
| 170 | UBE2S |  |  | 887 | -0.815 | -0.3359 | Yes |
| 171 | H2AFZ |  |  | 893 | -0.849 | -0.3362 | Yes |
| 172 | RAD51AP1 |  |  | 894 | -0.853 | -0.3297 | Yes |
| 173 | KPNA2 |  |  | 895 | -0.858 | -0.3232 | Yes |
| 174 | DBF4 |  |  | 896 | -0.864 | -0.3166 | Yes |
| 175 | SMC2 |  |  | 897 | -0.867 | -0.3100 | Yes |
| 176 | CENPE |  |  | 899 | -0.873 | -0.3046 | Yes |
| 177 | PBK |  |  | 900 | -0.880 | -0.2979 | Yes |
| 178 | SMC4 |  |  | 902 | -0.886 | -0.2925 | Yes |
| 179 | DUT |  |  | 903 | -0.887 | -0.2857 | Yes |
| 180 | MIS18BP1 |  |  | 904 | -0.889 | -0.2790 | Yes |
| 181 | CKS1B |  |  | 909 | -0.912 | -0.2774 | Yes |
| 182 | HJURP |  |  | 910 | -0.921 | -0.2704 | Yes |
| 183 | CKS2 |  |  | 911 | -0.922 | -0.2634 | Yes |
| 184 | GTSE1 |  |  | 912 | -0.941 | -0.2562 | Yes |
| 185 | MYBL2 |  |  | 914 | -0.959 | -0.2502 | Yes |
| 186 | CLSPN |  |  | 916 | -0.964 | -0.2442 | Yes |
| 187 | TYMS |  |  | 917 | -0.965 | -0.2368 | Yes |
| 188 | CENPU |  |  | 918 | -0.971 | -0.2294 | Yes |
| 189 | PRC1 |  |  | 919 | -0.980 | -0.2219 | Yes |
| 190 | MAD2L1 |  |  | 921 | -0.990 | -0.2157 | Yes |
| 191 | UBE2T |  |  | 922 | -0.994 | -0.2081 | Yes |
| 192 | SPC25 |  |  | 924 | -1.033 | -0.2016 | Yes |
| 193 | HIST1H1E |  |  | 925 | -1.049 | -0.1936 | Yes |
| 194 | ESCO2 |  |  | 926 | -1.067 | -0.1854 | Yes |
| 195 | HIST1H2AH |  |  | 927 | -1.078 | -0.1772 | Yes |
| 196 | UBE2C |  |  | 928 | -1.108 | -0.1687 | Yes |
| 197 | NUSAP1 |  |  | 929 | -1.112 | -0.1602 | Yes |
| 198 | TPX2 |  |  | 930 | -1.124 | -0.1517 | Yes |
| 199 | NCAPG |  |  | 931 | -1.142 | -0.1429 | Yes |
| 200 | ASPM |  |  | 932 | -1.146 | -0.1342 | Yes |
| 201 | ATAD2 |  |  | 933 | -1.162 | -0.1253 | Yes |
| 202 | HIST2H2AC |  |  | 934 | -1.162 | -0.1164 | Yes |
| 203 | CENPF |  |  | 935 | -1.200 | -0.1073 | Yes |
| 204 | HIST1H1C |  |  | 936 | -1.220 | -0.0979 | Yes |
| 205 | HMGB2 |  |  | 937 | -1.238 | -0.0885 | Yes |
| 206 | CDK1 |  |  | 938 | -1.248 | -0.0790 | Yes |
| 207 | RRM2 |  |  | 940 | -1.403 | -0.0696 | Yes |
| 208 | MKI67 |  |  | 941 | -1.407 | -0.0589 | Yes |
| 209 | TOP2A |  |  | 942 | -1.417 | -0.0480 | Yes |
| 210 | HIST1H1D |  |  | 944 | -1.810 | -0.0356 | Yes |
| 211 | HIST1H1B |  |  | 945 | -1.903 | -0.0210 | Yes |
| 212 | HIST1H4C |  |  | 946 | -2.752 | -0.0000 | Yes |
Table: GSEA details [plain text format]

  

Fig 2: FELDMAN\_2020\_S-G2: Random ES distribution      
 Gene set null distribution of ES for **FELDMAN\_2020\_S-G2**
